# Peri-Umbilicated Pseudopustules in Mpox

**DOI:** 10.1093/omcr/omag021

**Published:** 2026-03-23

**Authors:** Nitin Gupta, Shantala GB, Rima Sahay, Kumar Singh, Pothumarthy Venkata Swathikiran, Muralidhar Varma, Tirlangi Praveen Kumar

**Affiliations:** Department of Infectious Diseases, Kasturba Medical College, Manipal Academy of Higher Education, Madhavnagar, Manipal 576104, Karnataka, India; Department of Microbiology, Bangalore Medical College & Research Institute, Bengaluru 560002, Karnataka, India; Indian Council of Medical Research-National Institute of Virology, Pune 411021, Maharashtra, India; Department of Microbiology, Bangalore Medical College & Research Institute, Bengaluru 560002, Karnataka, India; Department of Infectious Diseases, Kasturba Medical College, Manipal Academy of Higher Education, Madhavnagar, Manipal 576104, Karnataka, India; Department of Infectious Diseases, Kasturba Medical College, Manipal Academy of Higher Education, Madhavnagar, Manipal 576104, Karnataka, India; Department of Infectious Diseases, Kasturba Medical College, Manipal Academy of Higher Education, Madhavnagar, Manipal 576104, Karnataka, India

**Keywords:** mpox, monkeypox, pseudopustules, genital ulcer, viral exanthem, emerging infections

## Abstract

Mpox is an emerging zoonotic infection that has shown sustained human-to-human transmission since 2022, often through sexual contact networks. We report a case of mpox in a 43-year-old Indian-origin man residing in the United Arab Emirates who presented with fever, sore throat, and a painful genital ulcer, followed by widespread vesiculo-pseudopustular lesions. Examination revealed firm, well-circumscribed, peri-umbilicated pseudopustules, distinctive lesions lacking purulent content. Laboratory investigations were unremarkable, and a polymerase chain reaction test for the Monkeypox virus, conducted on throat and skin swabs, confirmed the diagnosis. The patient was managed with supportive care and recovered uneventfully. This case highlights the characteristic pseudopustular morphology of mpox, which serves as a key diagnostic clue distinguishing it from other genital and vesicular eruptions, and underscores the need for clinicians to be aware of this condition amid evolving global epidemiology.

## Introduction

Mpox (formerly known as monkeypox) is an emerging zoonotic infection caused by the *Monkeypox virus*, an orthopoxvirus closely related to variola and vaccinia viruses [1]. Historically confined to Central and West Africa, mpox has re-emerged since 2022 with sustained human-to-human transmission across multiple continents, particularly affecting sexual networks of men who have sex with men (MSM) [1, 2]. This represents a major epidemiological shift from its earlier sporadic, zoonotically transmitted form [1]. Clinically, mpox is characterized by fever, lymphadenopathy, and a vesiculo-pustular rash that progresses synchronously [3]. However, recent outbreaks have revealed atypical manifestations, including localized genital ulcers, peri-anal lesions, and mucosal involvement that may mimic other sexually transmitted infections [3]. Here, we describe a case of laboratory-confirmed mpox in an Indian-origin male residing in the United Arab Emirates (UAE), highlighting the characteristic morphology of pseudopustular lesions, which serve as a key diagnostic clue distinguishing mpox from other vesicopustular dermatoses.

## Case report

A 43-year-old Indian-origin man residing in the UAE presented with a 5-day history of fever, sore throat, and a painful genital ulcer. The patient denied being a man who has sex with men, but reported close contact with another individual who later tested positive for mpox. Over the subsequent days, he developed multiple vesiculo-pseudopustular lesions, initially localized to the genital area and later spreading to the trunk and extremities.

**Figure 1:**
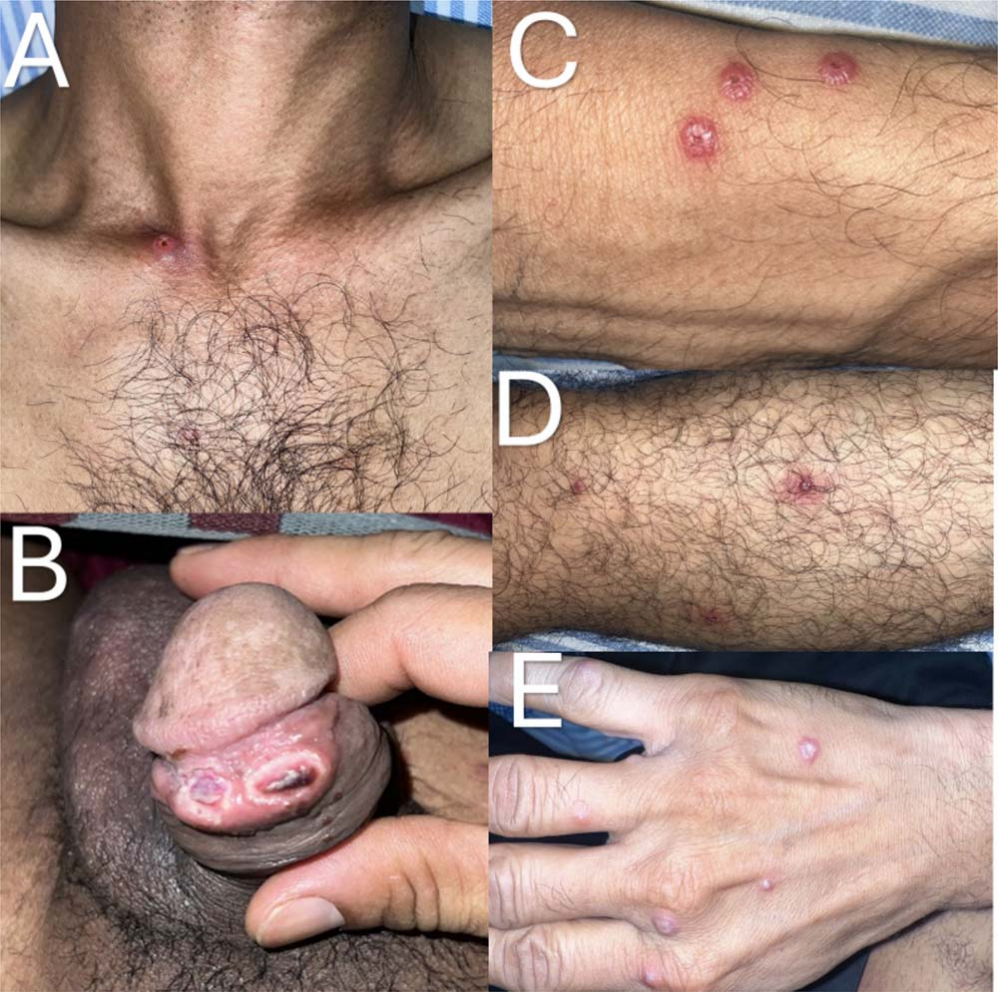
Characteristic pseudopustular lesions in mpox. (A) Ulcerated nodule over the upper chest. (B) Umbilicated erosions and ulcers on the glans penis and prepuce. (C–E) well-circumscribed, firm, peri-umbilicated pseudopustules with central crusting on the forearm (C), leg (D), and dorsum of the hand (E).

On admission, the patient was febrile (38.5°C) but hemodynamically stable. Physical examination revealed oral and genital ulcers, a maculopapular rash over the trunk, and numerous firm, well-circumscribed, peri-umbilicated pseudopustular lesions on the extremities (Fig. 1). These lesions lacked true purulent material, distinguishing them from typical pustules. Bilateral axillary and inguinal lymphadenopathy were palpable and mildly tender.

Laboratory investigations revealed a normal complete blood count and mild elevation of transaminases. Serologic tests for HIV, syphilis, hepatitis B, and hepatitis C were negative. Bacterial cultures from blood and lesion swabs yielded no growth. Given the epidemiological exposure and characteristic lesion morphology, mpox was suspected. Swabs from the throat and skin lesions were sent for PCR testing for the Monkeypox virus, which returned a positive result, confirming the diagnosis.

The patient was managed with supportive therapy, including antipyretics, topical antiseptics, and hydration, along with strict isolation precautions. No antiviral therapy (tecovirimat or brincidofovir) was administered, as the illness was self-limiting. Over the course of two weeks, the lesions crusted and resolved without scarring. The patient remained clinically stable and was discharged upon resolution of symptoms and completion of the isolation period.

## Discussion

The re-emergence of mpox in non-endemic regions underscores a paradigm shift in its transmission dynamics, from sporadic zoonotic spillover to sustained human-to-human transmission [1]. The predominant *Clade IIb* virus circulating globally since 2022 has demonstrated efficient transmission through close physical and sexual contact, particularly within interconnected sexual networks [4, 5]. This shift has led to changes not only in the epidemiology of the disease but also in its clinical spectrum and pattern of presentation.

Traditionally, mpox lesions evolve sequentially from macules to papules, vesicles, pustules, and crusts [3]. However, recent outbreaks have revealed considerable morphological variability. The hallmark finding in this case was the presence of pseudopustules, firm, umbilicated lesions that lack true purulent content, reflecting dermal edema and necrosis rather than suppuration [3]. Recognition of this morphology is critical, as it helps distinguish mpox from other vesiculo-pustular dermatoses [6]. Herpes simplex virus typically presents with grouped, fluid-filled vesicles on an erythematous base that rapidly ulcerate, whereas varicella-zoster virus produces pleomorphic lesions that evolve asynchronously [6]. Molluscum contagiosum, on the other hand, presents as smaller umbilicated papules with central keratin plugs and minimal inflammation [6]. In sexually active individuals, genital ulcers caused by herpes simplex virus, syphilis, or chancroid can further complicate the clinical picture [6]. Therefore, integrating lesion morphology with epidemiological context, such as recent exposure to a confirmed case or travel to an endemic area, remains key for timely clinical suspicion and diagnosis.

While specific antivirals such as tecovirimat showed promise initially, a subsequent randomised controlled trial has shown that it was ineffective in reducing the number of days to lesion resolution [7]. Early recognition, isolation, and appropriate infection control measures are essential to interrupt transmission. This case reinforces the importance of clinician awareness of the atypical and evolving presentations of mpox, especially the distinctive pseudopustular morphology, which can serve as an early diagnostic clue before confirmatory molecular testing is available.

## CRediT statement

SGB, RS, and KS were involved in the molecular diagnosis. NG, PVS, MV, and TPK contributed to the clinical management. NG drafted the manuscript. All authors reviewed the manuscript and provided critical revisions.
